# Bias caused by sampling error in meta-analysis with small sample sizes

**DOI:** 10.1371/journal.pone.0204056

**Published:** 2018-09-13

**Authors:** Lifeng Lin

**Affiliations:** Department of Statistics, Florida State University, Tallahassee, United States of America; Indiana University Bloomington, UNITED STATES

## Abstract

**Background:**

Meta-analyses frequently include studies with small sample sizes. Researchers usually fail to account for sampling error in the reported within-study variances; they model the observed study-specific effect sizes with the within-study variances and treat these sample variances as if they were the true variances. However, this sampling error may be influential when sample sizes are small. This article illustrates that the sampling error may lead to substantial bias in meta-analysis results.

**Methods:**

We conducted extensive simulation studies to assess the bias caused by sampling error. Meta-analyses with continuous and binary outcomes were simulated with various ranges of sample size and extents of heterogeneity. We evaluated the bias and the confidence interval coverage for five commonly-used effect sizes (i.e., the mean difference, standardized mean difference, odds ratio, risk ratio, and risk difference).

**Results:**

Sampling error did not cause noticeable bias when the effect size was the mean difference, but the standardized mean difference, odds ratio, risk ratio, and risk difference suffered from this bias to different extents. The bias in the estimated overall odds ratio and risk ratio was noticeable even when each individual study had more than 50 samples under some settings. Also, Hedges’ *g*, which is a bias-corrected estimate of the standardized mean difference within studies, might lead to larger bias than Cohen’s *d* in meta-analysis results.

**Conclusions:**

Cautions are needed to perform meta-analyses with small sample sizes. The reported within-study variances may not be simply treated as the true variances, and their sampling error should be fully considered in such meta-analyses.

## Introduction

Systematic reviews and meta-analyses have become important tools to synthesize results from various studies in a wide range of areas, especially in clinical and epidemiological research [1–3]. Sampling error is a critical issue in meta-analyses. On the one hand, it impacts the evaluation of heterogeneity between studies. For example, the popular heterogeneity measure *I*^2^ statistic is supposed to quantify the proportion of variation due to heterogeneity rather than sampling error [4–6]; if sampling error increases, the *I*^2^ tends to decrease, leading to a conclusion of more homogeneous studies. More troublesome, within-study sampling error may affect the derivation underlying *I*^2^ to such an extent that the interpretation of *I*^2^ is challenged [7]. On the other hand, sampling error may threaten the validity of a meta-analysis. The most popular meta-analysis method usually models the observed effect size in each study as a normally distributed random variable and treats the observed sample variance as if it was the true variance [8, 9]. It accounts for sampling error in the point estimate of the treatment effect within each study, but it ignores sampling error in the observed variance. This method is generally valid when the number of samples within each collected study is large: the large-sample statistical properties, such as the central limit theory and the delta method, guarantee that the distribution approximation performs well. However, ignoring sampling error in within-study variances has caused some misunderstandings about basic quantities in meta-analyses, especially when some studies have few samples. For example, the famous *Q* test for homogeneity does not exactly follow the chi-squared distribution due to such sampling error [10], and this problem may subvert *I*^2^ [7].

One important purpose of performing meta-analyses is to increase precision as well as to reduce bias for the conclusions of systematic reviews [11]. For this reason, the PRISMA statement [12] recommends researchers to report both the risks of bias within individual studies and also between studies. The bias within individual studies often relates to the studies’ quality [13]. Also, certain measures have been designed to reduce bias in study-level estimates. For example, Hedges’ *g* is considered less biased than Cohen’s *d* within studies when the effect size is the standardized mean difference (page 81 in Hedges and Olkin [14]). The bias between studies is usually introduced by publication bias or selective reporting [15–20]. Besides the bias in point estimates of treatment effects, sampling error also produces bias in the variance of the overall weighted mean estimate under the fixed-effect setting [21, 22]. Under the random-effects setting, the well-known DerSimonian–Laird estimator of the between-study variance may also have considerable bias, especially when sample sizes are small [23, 24]. Moreover, the between-study bias in the treatment effect estimates, such as publication bias, may implicate other parameters in a meta-analysis, including the between-study variance [25]. The bias in variance estimates can seriously impact the precision of the meta-analysis results.

This article focuses on the performance of meta-analyses with small sample sizes, where the sampling error in the observed within-study variances may not be ignored. Throughout this article, we refer to sample size as the number of participants in an individual study, instead of the number of studies in a meta-analysis. Studies with small or moderate sample sizes are fairly common in meta-analyses [26], especially when the treatments are expensive and the enrollments of participants are limited by studies’ budgets. We demonstrate a type of bias in meta-analysis results that is completely due to sampling error; it has received relatively less attention in the existing literature compared with other types of bias [27–30]. Such bias is mainly caused by the association between the observed study-specific effect sizes *y*_*i*_ and their within-study variances $s_{i}^{2}$. This association may exist even in the absence of publication bias or selective reporting [31, 32]. When one uses the true variances instead of the estimated variances, the association may still be present for certain effect sizes, e.g., the (log) odds ratio.

If each study’s result is unbiased and its marginal expectation equals to some overall treatment effect *θ*, then a naïve argument for the unbiasedness of the overall effect estimate in a meta-analysis, $\hat{\theta }=\frac{\sum w_{i}y_{i}}{\sum w_{i}}$, is that $E[\hat{\theta }]=\frac{\sum w_{i}E[y_{i}]}{\sum w_{i}}=\theta$, where *w*_*i*_ is the weight of each study. The weight is usually the inverse of the within-study variance or the marginal variance incorporating heterogeneity between studies. However, this equation treats the weights *w*_*i*_ as fixed values, while in practice they are estimates subject to sampling error. The association between the observed effect sizes and their estimated within-study variances may be strong when the sample sizes are small, so the expectation of the overall estimate in the meta-analysis may not be directly derived without the information about such association, and its unbiasedness is largely unclear [33]. In addition, when the sample sizes are small, the sampling error in the observed within-study variances and the estimated between-study variance may be large, so the confidence interval (CI) of the overall estimate may be poor with coverage probability much lower than the nominal level.

In the following sections, we will review five common effect sizes for continuous and binary outcomes, explain how small sample sizes may introduce bias in meta-analyses, and evaluate such bias using extensive simulation studies.

## Methods

### Meta-analyses with continuous outcomes

Suppose that a meta-analysis contains *N* studies, and each study compares a treatment group with a control group. Denote *n*_*i*0_ and *n*_*i*1_ as the sample sizes in the control and treatment groups in study *i*. The continuous outcome measures of the participants in each group are assumed to follow normal distributions. The population means of the two groups in study *i* are *μ*_*i*0_ and *μ*_*i*1_, and the sample means are denoted as ${\overset{-}{y}}_{i0}$ and ${\overset{-}{y}}_{i1}$ accordingly. The variances of the samples in the two groups are frequently assumed to be equal, denoted as $\sigma_{i}^{2}$; see, e.g., page 76 in Hedges and Olkin [14] and page 224 in Cooper et al. [34]. The $\sigma_{i}^{2}$ is estimated as the pooled sample variance $s_{iP}^{2}=\frac{(n_{i0}-1)S_{i0}^{2}+(n_{i1}-1)S_{i1}^{2}}{n_{i0}+n_{i1}-2}$, where $s_{i0}^{2}$ and $s_{i1}^{2}$ are the sample variances in the control and treatment groups, respectively.

If the outcome measures have a meaningful scale and all studies in the meta-analysis are reported on the same scale, the mean difference (MD) between the two groups, i.e., Δ_*i*_ = *μ*_*i*1_ − *μ*_*i*0_, is often used as the effect size to measure treatment effect (page 224 in Cooper et al. [34]). We can obtain an estimate of the MD from each study, denoted as $y_{i}={\overset{-}{y}}_{i1}-{\overset{-}{y}}_{i0}$, and its estimated within-study variance is $s_{i}^{2}=\left(\frac{1}{n_{i0}}+\frac{1}{n_{i1}}\right)s_{iP}^{2}$. Traditional meta-analysis methods usually account for sampling error in the sample means *y*_*i*_ but ignore such error in the sample variances $s_{i}^{2}$; the within-study variances have been customarily treated as the true variances, which should be $(\frac{1}{n_{i0}}+\frac{1}{n_{i1}})\sigma_{i}^{2}$ [10]. However, accurate estimates of variances may require very large sample sizes; the sample variances $s_{i}^{2}$ may be far away from their true values when sample sizes are small. In the following context, we will treat the sample variances as random variables like the sample means, instead of the true variances.

Because the outcome measures are assumed to be normal, the sample means ${\overset{-}{y}}_{i0}$ and ${\overset{-}{y}}_{i1}$ are independent of the sample variances $s_{i0}^{2}$ and $s_{i1}^{2}$ (see page 218 in Casella and Berger [35]). Thus, the *y*_*i*_ and $s_{i}^{2}$ are independent in each study. Given that the observed MDs *y*_*i*_ are unbiased, such independence guarantees that the overall effect size estimate is unbiased in a fixed-effect meta-analysis (which assumes that the underlying true effect sizes Δ_*i*_ in all studies equal to a common value Δ), because $E\left[\frac{\sum y_{i}/s_{i}^{2}}{\sum 1/s_{j}^{2}}\right]=\sum E\left[y_{i}\right]E\left[\frac{1/s_{i}^{2}}{\sum 1/s_{j}^{2}}\right]=\Delta \sum E[\frac{1/s_{i}^{2}}{\sum 1/s_{j}^{2}}]=\Delta$. However, in a random-effects meta-analysis, each study’s weight is updated as $1/\left(s_{i}^{2}+{\hat{\tau }}^{2}\right)$ by incorporating an estimate of the between-study variance ${\hat{\tau }}^{2}$. The between-study variance *τ*^2^ can be estimated using many different methods [36], and its estimate depends on both *y*_*i*_ and $s_{i}^{2}$; therefore, *y*_*i*_ and the updated weight $1/\left(s_{i}^{2}+{\hat{\tau }}^{2}\right)$ may be correlated to some extents. The expectation of the weighted average cannot be split in the foregoing way, so the unbiasedness of the overall MD estimate is not guaranteed in a random-effects meta-analysis.

A more commonly-used effect size for continuous outcomes is the standardized mean difference (SMD), because this unit-free measure permits different scales in the collected studies and is deemed more comparable across studies (see Normand [8] and Chapter 3 in Grissom and Kim [37]). The true SMD in study *i* is $\theta_{i}=\frac{\mu_{i1}-\mu_{i0}}{\sigma_{i}}$. It is usually estimated as $y_{i}=\frac{{\overset{-}{y}}_{i1}-{\overset{-}{y}}_{i0}}{s_{iP}}$ by plugging in the sample means and the pooled variance, and is often referred to as Cohen’s *d* (see page 66 in Cohen [38]). If we define a constant *q*_*i*_ = *n*_*i*0_*n*_*i*1_/(*n*_*i*0_ + *n*_*i*1_), multiply Cohen’s *d* by $\sqrt{q_{i}}$, and express it as $\sqrt{q_{i}}y_{i}=\frac{\sqrt{q_{i}}({\overset{-}{y}}_{i1}-{\overset{-}{y}}_{i0})/\sigma_{i}}{s_{iP}/\sigma_{i}}$, then the numerator follows a normal distribution with variance 1, and the denominator is the square root of a chi-squared random variable $(n_{i0}+n_{i1}-2)s_{ip}^{2}/\sigma_{i}^{2}$ divided by its degrees of freedom *n*_*i*0_ + *n*_*i*1_ − 2 [35]. Also, the numerator and denominator are independent. Therefore, strictly speaking, Cohen’s *d* (multiplied by the constant $\sqrt{q_{i}}$) follows a *t*-distribution, although it is approximated as a normal distribution in nearly all applications. If the true effect size is non-zero, the *t*-distribution is noncentral. The exact within-study variance of Cohen’s *d* can be derived as a complicated form of gamma functions [39], but researchers usually use some simpler forms to approximate it. Different approximation forms for the within-study variance of Cohen’s *d* are given in several books on meta-analyses; see, e.g., page 80 in Hedges and Olkin [14], page 226 in Cooper et al. [34], and page 290 in Egger et al. [40]. This article approximates it as $s_{i}^{2}=\frac{1}{n_{i0}}+\frac{1}{n_{i1}}+\frac{y_{i}^{2}}{2(n_{i0}+n_{i1}-2)}$. As $s_{i}^{2}$ depends on *y*_*i*_, they are correlated. The correlation may increase as the sample size decreases, because the coefficient of $y_{i}^{2}$ in the formula of $s_{i}^{2},\;\frac{1}{2(n_{i0}+n_{i1}-2)}$, increases.

Furthermore, it is well-known that Cohen’s *d* is a biased estimate of the SMD. The bias is around $\frac{3\theta_{i}}{4(n_{i0}+n_{i1})-9}$ (page 80 in Hedges and Olkin [14]); and it reduces toward zero as the sample sizes increase. When the sample sizes are small, a bias-corrected estimate, called Hedges’ *g*, is usually adopted [41]. It is calculated as $y_{i}=[1-\frac{3}{4(n_{i0}+n_{i1})-9}]\cdot \frac{{\overset{-}{y}}_{i1}-{\overset{-}{y}}_{i0}}{s_{iP}}$ with an estimated variance $s_{i}^{2}=\frac{1}{n_{i0}}+\frac{1}{n_{i1}}+\frac{y_{i}^{2}}{2(n_{i0}+n_{i1})}$ (page 86 in Hedges and Olkin [14]). Like Cohen’s *d*, the observed data *y*_*i*_ and $s_{i}^{2}$ are also correlated when using Hedges’ *g* as the effect size. Therefore, even if Hedges’ *g* is (nearly) unbiased within each individual study, the overall SMD estimate in the meta-analysis may be still biased due to the correlation between *y*_*i*_ and $s_{i}^{2}$.

### Meta-analyses with binary outcomes

Suppose a 2 × 2 table is available from each collected study in a meta-analysis with a binary outcome. Denote *n*_*i*00_ and *n*_*i*01_ as the numbers of participants without and with an event in the control group, respectively; *n*_*i*10_ and *n*_*i*11_ are the data cells in the treatment group. The sample sizes in the control and treatment groups are *n*_*i*0_ = *n*_*i*00_ + *n*_*i*01_ and *n*_*i*1_ = *n*_*i*10_ + *n*_*i*11_. Also, denote *p*_*i*0_ and *p*_*i*1_ as the population event rates in the two groups.

The odds ratio (OR) is frequently used to measure treatment effect for a binary outcome [42]; its true value in study *i* is ${OR}_{i}=\frac{p_{i1}/\left(1-p_{i1}\right)}{p_{i0}/\left(1-p_{i0}\right)}$. Using the four data cells in the 2×2 table, the OR is estimated as ${\hat{OR}}_{i}=\frac{n_{i00}n_{i11}}{n_{i01}n_{i10}}$. The ORs are usually combined on a logarithmic scale in meta-analyses, because the distribution of the estimated log OR, $y_{i}=\log\;{\hat{OR}}_{i}$ is better approximated by a normal distribution. The within-study variance of *y*_*i*_ is estimated as $s_{i}^{2}=\frac{1}{n_{i00}}+\frac{1}{n_{i01}}+\frac{1}{n_{i10}}+\frac{1}{n_{i11}}$. Besides the OR, the risk ratio (RR) and risk difference (RD) are also popular effect sizes. The underlying true RR in study *i* is RR_*i*_ = *p*_*i*1_/*p*_*i*0_, and it is also combined on the log scale in meta-analyses like the OR. The log RR is estimated as $y_{i}=\log\frac{n_{i11}/n_{i1}}{n_{i01}/n_{i0}}$, and its within-study variance is estimated as $s_{i}^{2}=\frac{1}{n_{i01}}+\frac{1}{n_{i11}}-\frac{1}{n_{i0}}-\frac{1}{n_{i1}}$. Moreover, the underlying true RD in study *i* is RD_*i*_ = *p*_*i*1_ − *p*_*i*0_, estimated as $y_{i}=\frac{n_{i11}}{n_{i1}}-\frac{n_{i01}}{n_{i0}}$ with an estimated within-study variance $s_{i}^{2}=\frac{n_{i00}n_{i01}}{n_{i0}^{3}}+\frac{n_{i10}n_{i11}}{n_{i1}^{3}}$. When the sample sizes are small, some data cells may be zero even if the event is not rare. If a 2 × 2 table contains zero cells, a fixed value of 0.5 is often added to each data cell to reduce bias and avoid computational error (see page 521 in the *Cochrane Handbook for Systematic Reviews of Interventions* [43] and many other papers [44–46]), although this continuity correction may not be optimal in some cases [47–50].

Like the SMD for continuous outcomes, the distributions of the sample log OR, log RR, and RD are approximated as normal distributions in conventional meta-analysis methods. Also, because both *y*_*i*_ and $s_{i}^{2}$ depend on the four cells of 2 × 2 tables for all three effect sizes, they are intrinsically correlated.

### Simulation studies

We conducted simulation studies to investigate the impact of sampling error on meta-analyses with small sample sizes. The number of studies in a simulated meta-analysis was set to *N* = 5, 10, 20, and 50. We first generated the sample size within each study *n*_*i*_ from a uniform distribution *U*(5, 10), then we gradually increased it by sampling it from *U*(10, 20), *U*(20, 30), *U*(30, 50), *U*(50, 100), *U*(100, 500), and *U*(500, 1000). These sample sizes *n*_*i*_ were generated anew for each simulated meta-analysis. The control/treatment allocation ratio was set to 1:1 in all studies, which is common in real-world applications. Specifically, $n_{i0}=\lceil \frac{n_{i}}{2}\rceil$ participants were assigned to the control group and $n_{i1}=n_{i}-\lceil \frac{n_{i}}{2}\rceil$ participants were assigned to the treatment group, where ⌈*x*⌉ represents an integer that is greater than or equal to *x*.

When the outcome was continuous, we simulated meta-analyses based on the MD and the SMD. For the MD, each participant’s outcome measure was sampled from $N(\mu_{i0},\sigma_{i}^{2})$ in the control group or $N(\mu_{i0}+\Delta_{i},\sigma_{i}^{2})$ in the treatment group. Without loss of generality, the baseline effect *μ*_*i*0_ of study *i* was generated from *N*(0,1). The study-specific standard deviation *σ*_*i*_ was sampled from *U*(1,5), and it was generated anew for each simulated meta-analysis. The mean difference Δ_*i*_ was sampled from *N*(Δ,*τ*^2^). The overall MD Δ was set to 0, 0.5, 1, 2, and 5, and the between-study standard deviation *τ* was set to 0, 0.5, and 1. For the SMD, each participant’s outcome measure was also generated using the foregoing setting within each study, but the SMD *θ*_*i*_ = Δ_*i*_/*σ*_*i*_, not the mean difference Δ_*i*_, was sampled from the normal distribution: *θ*_*i*_ ~ *N*(*θ*,*τ*^2^). The overall SMD *θ* was set to 0, 0.2, 0.5, 0.8, and 1 to represent different magnitudes of effect size. The between-study standard deviation *τ* was 0, 0.2, and 0.5. Both Cohen’s *d* and Hedges’ *g* were used to estimate the SMD.

When the outcome was binary, we first simulated meta-analyses based on the OR. The event numbers *n*_*i*01_ and *n*_*i*11_ in the control and treatment groups were sampled from Binomial(*n*_*i*0_,*p*_*i*0_) and Binomial(*n*_*i*1_,*p*_*i*1_), respectively. The event rate in the control group *p*_*i*0_ was sampled from *U*(0.3, 0.7) representing a fairly common event [32], and it was generated anew for each meta-analysis. The event rate in the treatment group *p*_*i*1_ was calculated using *p*_*i*0_ and the study-specific log OR *θ*_*i*_; specifically, $p_{i1}={\left[1+e^{-\theta_{i}}\;\left(1-p_{i0}\right)/p_{i0}\right]}^{-1}$. The study-specific log OR *θ*_*i*_ was sampled from *N*(*θ*,*τ*^2^), where the overall log OR *θ* was set to 0, 0.2, 0.4, 1, and 1.5, and the between-study standard deviation *τ* was 0, 0.2, and 0.5. In addition to the OR, we also generated meta-analyses based on the RR and RD. The event numbers were similarly sampled from binomial distributions and the *p*_*i*0_ was from *U*(0.3,0.7). However, for the log RR and the RD, we considered only the fixed-effect setting with all study-specific effect sizes *θ*_*i*_ equal to a common value *θ*. Specifically, if the effect size was the log RR, the event rate in the treatment group was *p*_*i*1_ = *e*^*θ*^*p*_*i*0_, where the true log RR *θ* was set to 0 and 0.3 to guarantee that *p*_*i*1_ was between 0 and 1. If the effect size was the RD, *p*_*i*1_ = *p*_*i*0_ +*θ*, where the true RD *θ* was set to 0 and 0.2 to guarantee that *p*_*i*1_ was between 0 and 1. The random-effects setting was not considered for the log RR and RD because it may lead to improper *p*_*i*1_’s beyond the [0, 1] range. We could successfully generate meta-analyses by truncating such improper *p*_*i*1_’s and constraining them to be between 0 and 1; however, this constraint would produce bias, which cannot be distinguished from the bias caused by sampling error that is of primary interest in this article.

For each simulation setting above, 10,000 meta-analyses were generated. The random-effects model was applied to each simulated meta-analysis [51], even if some meta-analyses were generated under the fixed-effect setting with *τ* = 0. Thus, the produced CIs might be conservative. Also, the between-study variance was estimated using the popular method of moments by DerSimonian and Laird [24]. The restricted maximum likelihood method may be a better choice [8, 52, 53], but it is more computationally difficult and its solution did not converge in a noticeable number of our simulated meta-analyses. Also, there are many other alternatives for estimating the between-study variance, such as the Paule–Mandel estimator, which may be recommended in certain situations [54, 55], while they have been used less frequently compared with the DerSimonian–Laird estimator so far. Therefore, we considered only the DerSimonian–Laird estimator for the between-study variance, which was sufficient to achieve this article’s purpose.

S2–S7 Files present the R code and results for the simulation studies.

## Results

**Fig 1:**
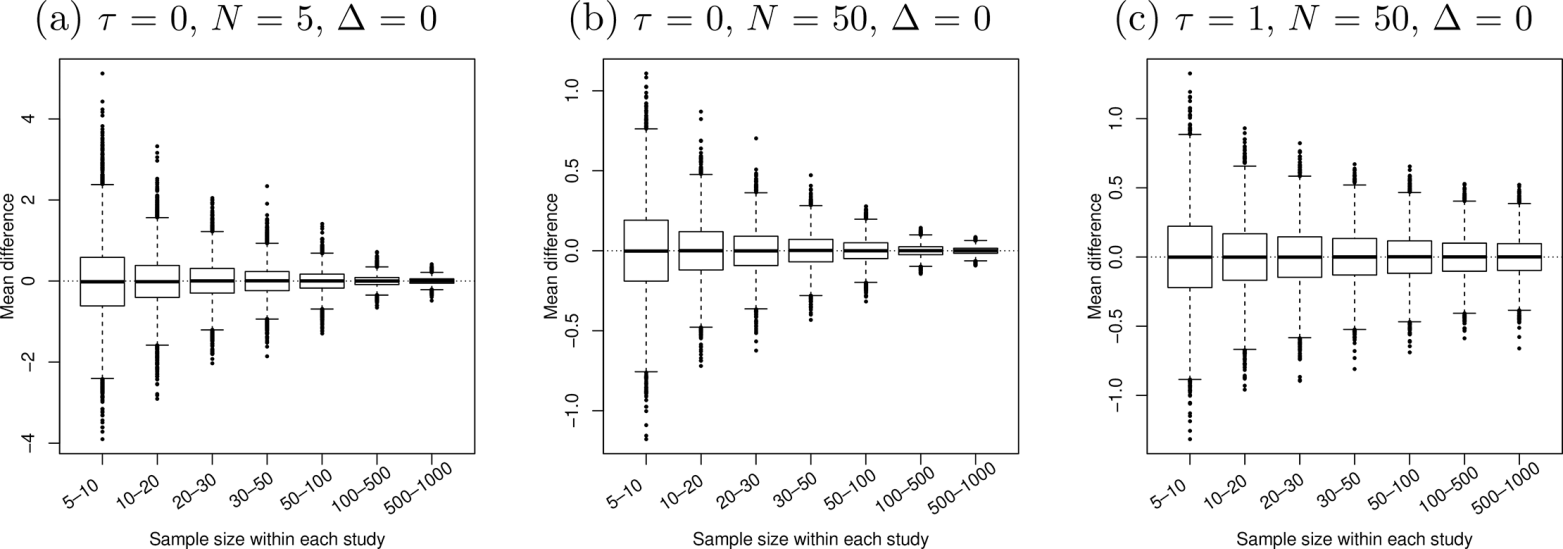
Boxplots of the estimated mean differences in 10,000 simulated meta-analyses. The true between-study standard deviation *τ* increased from 0 (panels a and b) to 1 (panel c). The number of studies in each meta-analysis *N* increased from 5 (panel a) to 50 (panels b and c). The true mean difference Δ (horizontal dotted line) was 0.

**Fig 2:**
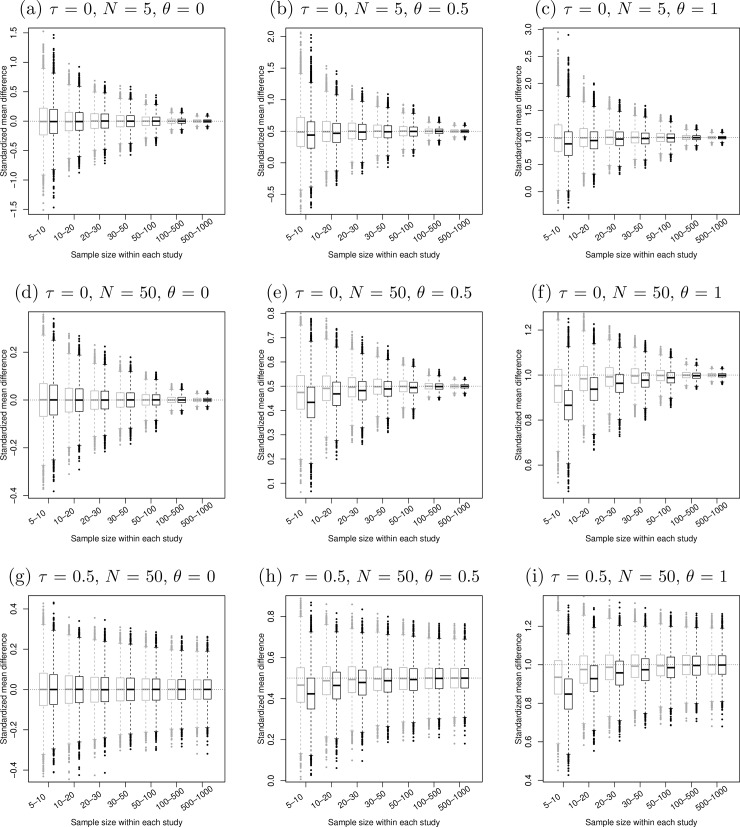
Boxplots of the estimated standardized mean differences in 10,000 simulated meta-analyses. For each sample size range on the horizontal axis, the left gray box was obtained using Cohen’s *d*, and the right black box was obtained using Hedges’ *g*. The true between-study standard deviation *τ* increased from 0 (upper and middle panels) to 0.5 (lower panels). The number of studies in each meta-analysis *N* increased from 5 (upper panels) to 50 (middle and lower panels). The true standardized mean difference *θ* (horizontal dotted line) increased from 0 (left panels) to 1 (right panels).

**Fig 3:**
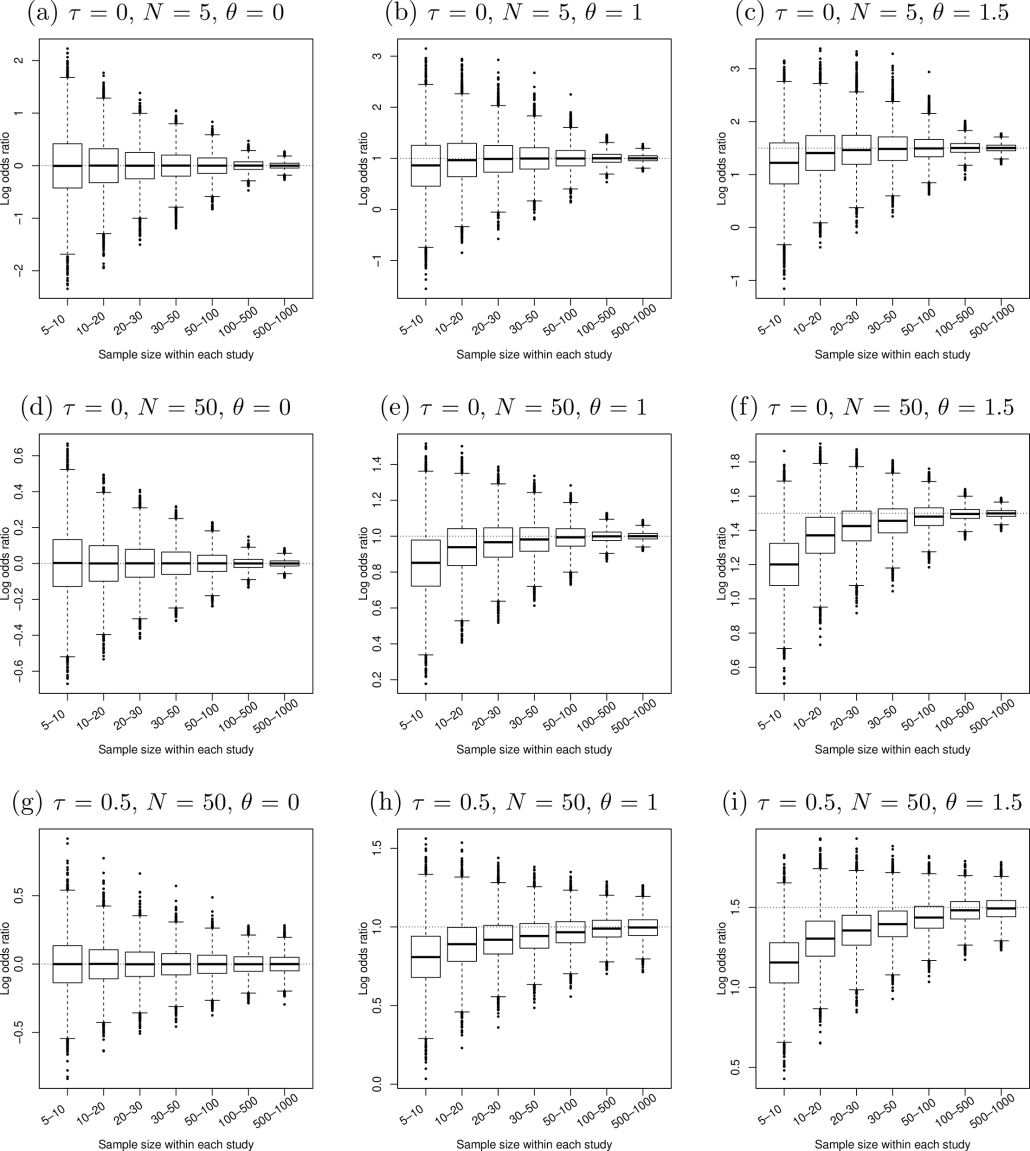
Boxplots of the estimated log odds ratios in 10,000 simulated meta-analyses. The true between-study standard deviation *τ* increased from 0 (upper and middle panels) to 0.5 (lower panels). The number of studies in each meta-analysis *N* increased from 5 (upper panels) to 50 (middle and lower panels). The true log odds ratio *θ* (horizontal dotted line) increased from 0 (left panels) to 1.5 (right panels).

**Fig 4:**
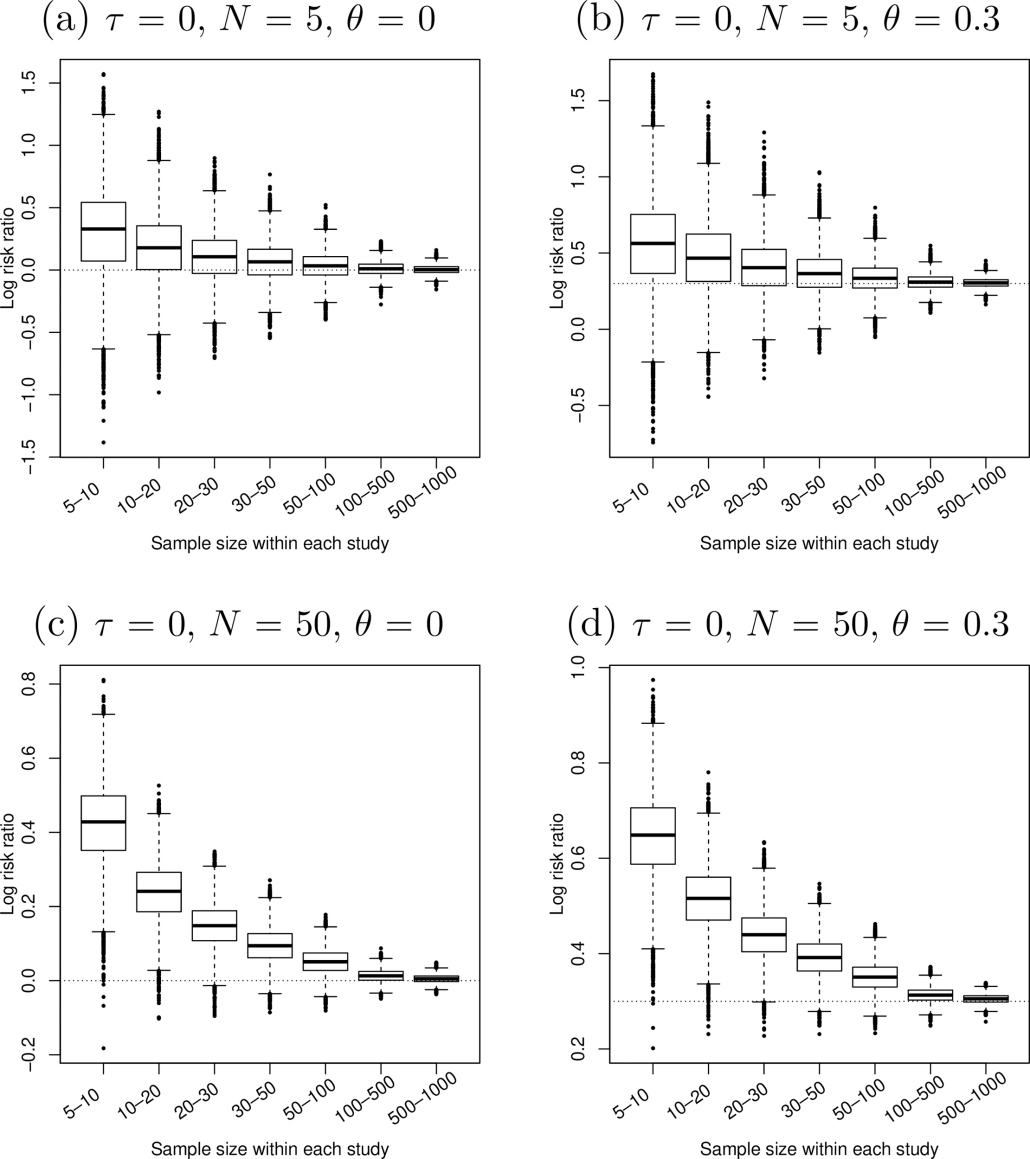
Boxplots of the estimated log risk ratios in 10,000 simulated meta-analyses. The true between-study standard deviation *τ* was 0 (i.e., the simulated studies were homogeneous). The number of studies in each meta-analysis *N* increased from 5 (upper panels) to 50 (lower panels). The true log risk ratio *θ* (horizontal dotted line) increased from 0 (left panels) to 0.3 (right panels).

**Fig 5:**
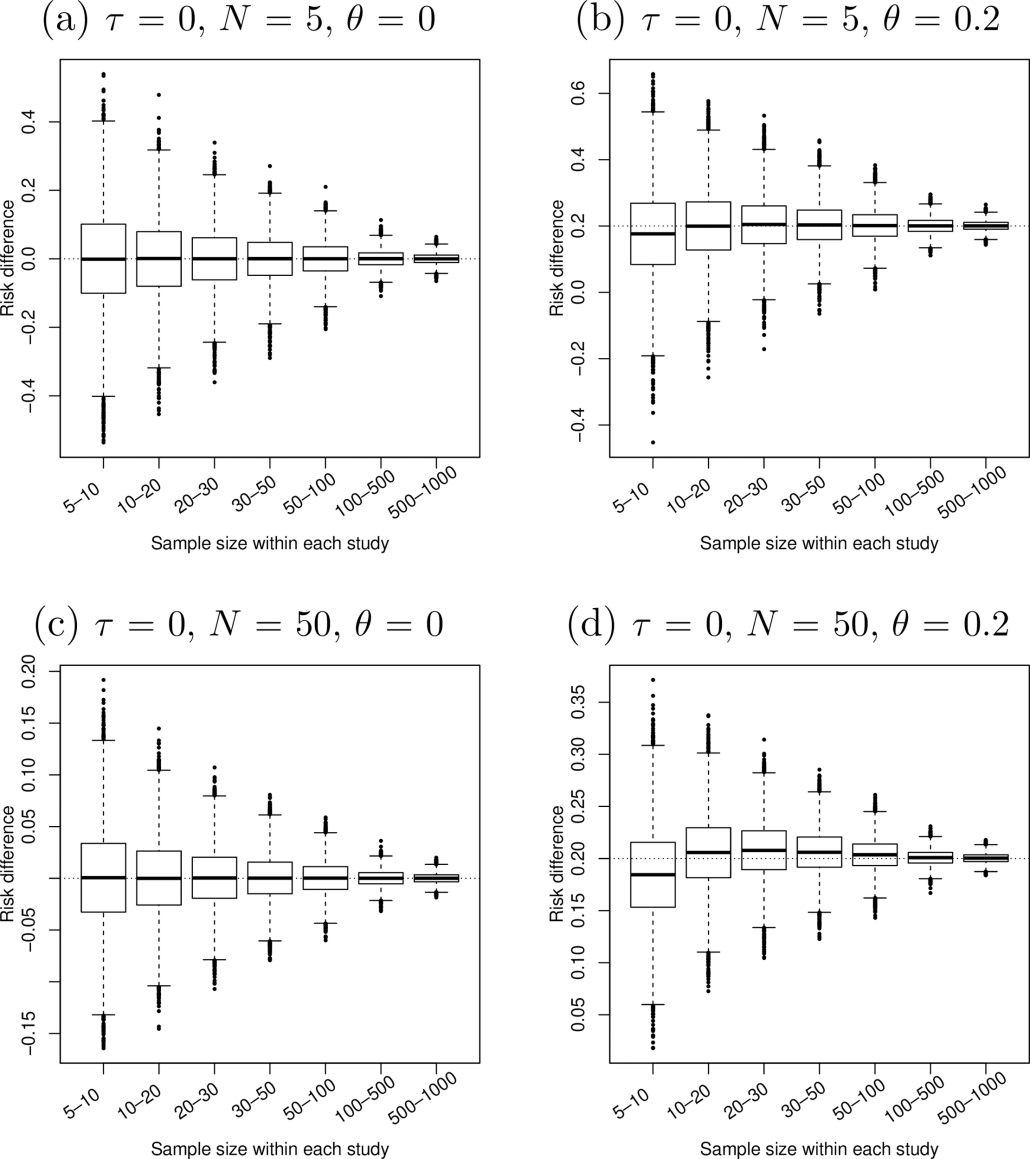
Boxplots of the estimated risk differences in 10,000 simulated meta-analyses. The true between-study standard deviation *τ* was 0 (i.e., the simulated studies were homogeneous). The number of studies in each meta-analysis *N* increased from 5 (upper panels) to 50 (lower panels). The true risk difference *θ* (horizontal dotted line) increased from 0 (left panels) to 0.2 (right panels).

Fig 1–5 present the boxplots of the estimated overall effect sizes in the 10,000 simulated meta-analyses for the MD, SMD (estimated by both Cohen’s *d* and Hedges’ *g*), log OR, log RR, and RD, respectively. In addition, Table 1 shows the bias of the estimates and Table 2 shows their 95% CIs’ coverage probabilities. When the number of studies in a meta-analysis increased from 5 to 50, the range of the estimated overall effect size shrank because their variances decreased. When the between-study heterogeneity increased in Fig 1–3, the middle and lower panels indicate that the box of the estimated overall effect sizes expanded vertically due to more heterogeneity in the meta-analyses.

**Table 1:** Bias of the estimated overall effect size in the simulation studies.

Setting	Sample size						
	5–10	10–20	20–30	30–50	50–100	100–500	500–1000
Mean difference:							
*τ* = 0, *N* = 5, Δ = 0	−0.01	−0.01	0.01	0.00	0.00	0.00	0.00
*τ* = 0, *N* = 50, Δ = 0	0.00	0.00	0.00	0.00	0.00	0.00	0.00
*τ* = 1, *N* = 50, Δ = 0	0.00	0.00	0.00	0.00	0.00	0.00	0.00
Standardized mean difference (Cohen’s *d*):							
*τ* = 0, *N* = 5, *θ* = 0	0.00	0.00	0.00	0.00	0.00	0.00	0.00
*τ* = 0, *N* = 5, *θ* = 0.5	0.00	0.00	0.00	0.00	0.00	0.00	0.00
*τ* = 0, *N* = 5, *θ* = 1	0.01	0.00	0.01	0.00	0.00	0.00	0.00
*τ* = 0, *N* = 50, *θ* = 0	0.00	0.00	0.00	0.00	0.00	0.00	0.00
*τ* = 0, *N* = 50, *θ* = 0.5	−0.02	−0.01	0.00	0.00	0.00	0.00	0.00
*τ* = 0, *N* = 50, *θ* = 1	−0.05	−0.02	−0.01	0.00	0.00	0.00	0.00
*τ* = 0.5, *N* = 50, *θ* = 0	0.00	0.00	0.00	0.00	0.00	0.00	0.00
*τ* = 0.5, *N* = 50, *θ* = 0.5	−0.03	−0.01	−0.01	0.00	0.00	0.00	0.00
*τ* = 0.5, *N* = 50, *θ* = 1	−0.06	−0.02	−0.01	−0.01	0.00	0.00	0.00
Standardized mean difference (Hedges’ *g*):							
*τ* = 0, *N* = 5, *θ* = 0	0.00	0.00	0.00	0.00	0.00	0.00	0.00
*τ* = 0, *N* = 5, *θ* = 0.5	−0.05	−0.03	−0.01	−0.01	0.00	0.00	0.00
*τ* = 0, *N* = 5, *θ* = 1	−0.10	−0.05	−0.02	−0.02	−0.01	0.00	0.00
*τ* = 0, *N* = 50, *θ* = 0	0.00	0.00	0.00	0.00	0.00	0.00	0.00
*τ* = 0, *N* = 50, *θ* = 0.5	−0.07	−0.03	−0.02	−0.01	−0.01	0.00	0.00
*τ* = 0, *N* = 50, *θ* = 1	−0.13	−0.06	−0.04	−0.02	−0.01	0.00	0.00
*τ* = 0.5, *N* = 50, *θ* = 0	0.00	0.00	0.00	0.00	0.00	0.00	0.00
*τ* = 0.5, *N* = 50, *θ* = 0.5	−0.08	−0.04	−0.02	−0.01	−0.01	0.00	0.00
*τ* = 0.5, *N* = 50, *θ* = 1	−0.15	−0.07	−0.04	−0.03	−0.01	0.00	0.00
Log odds ratio:							
*τ* = 0, *N* = 5, *θ* = 0	0.00	0.00	0.00	0.00	0.00	0.00	0.00
*τ* = 0, *N* = 5, *θ* = 1	−0.15	−0.03	0.00	0.00	0.00	0.00	0.00
*τ* = 0, *N* = 5, *θ* = 1.5	−0.30	−0.09	−0.02	−0.01	0.00	0.00	0.00
*τ* = 0, *N* = 50, *θ* = 0	0.00	0.00	0.00	0.00	0.00	0.00	0.00
*τ* = 0, *N* = 50, *θ* = 1	−0.15	−0.06	−0.04	−0.02	−0.01	0.00	0.00
*τ* = 0, *N* = 50, *θ* = 1.5	−0.30	−0.13	−0.07	−0.04	−0.02	0.00	0.00
*τ* = 0.5, *N* = 50, *θ* = 0	0.00	0.00	0.00	0.00	0.00	0.00	0.00
*τ* = 0.5, *N* = 50, *θ* = 1	−0.19	−0.11	−0.08	−0.06	−0.03	−0.01	0.00
*τ* = 0.5, *N* = 50, *θ* = 1.5	−0.35	−0.19	−0.14	−0.10	−0.06	−0.02	−0.01
Log risk ratio:							
*τ* = 0, *N* = 5, *θ* = 0	0.30	0.18	0.11	0.07	0.03	0.01	0.00
*τ* = 0, *N* = 5, *θ* = 0.3	0.26	0.17	0.11	0.07	0.04	0.01	0.00
*τ* = 0, *N* = 50, *θ* = 0	0.42	0.24	0.15	0.09	0.05	0.01	0.01
*τ* = 0, *N* = 50, *θ* = 0.3	0.35	0.22	0.14	0.09	0.05	0.01	0.01
Risk difference:							
*τ* = 0, *N* = 5, *θ* = 0	0.00	0.00	0.00	0.00	0.00	0.00	0.00
*τ* = 0, *N* = 5, *θ* = 0.2	−0.02	0.00	0.00	0.00	0.00	0.00	0.00
*τ* = 0, *N* = 50, *θ* = 0	0.00	0.00	0.00	0.00	0.00	0.00	0.00
*τ* = 0, *N* = 50, *θ* = 0.2	−0.02	0.01	0.01	0.01	0.00	0.00	0.00

**Table 2:** Coverage probability (in percentage, %) of the estimated overall effect size’s 95% confidence interval in the simulation studies.

Setting	Sample size						
	5–10	10–20	20–30	30–50	50–100	100–500	500–1000
Mean difference:							
*τ* = 0, *N* = 5, Δ = 0	92.0	94.7	95.4	96.0	95.9	96.1	96.6
*τ* = 0, *N* = 50, Δ = 0	92.9	94.3	95.0	95.2	95.8	96.2	96.0
*τ* = 1, *N* = 50, Δ = 0	93.8	93.6	93.8	94.5	94.2	94.1	94.2
Standardized mean difference (Cohen’s *d*):							
*τ* = 0, *N* = 5, *θ* = 0	97.1	96.4	96.4	96.6	96.2	96.2	96.2
*τ* = 0, *N* = 5, *θ* = 0.5	97.2	96.6	96.3	96.4	96.3	96.2	96.3
*τ* = 0, *N* = 5, *θ* = 1	97.2	96.7	96.4	96.4	96.2	96.1	96.4
*τ* = 0, *N* = 50, *θ* = 0	97.1	96.2	96.2	95.9	96.1	95.8	95.8
*τ* = 0, *N* = 50, *θ* = 0.5	96.5	96.2	95.9	95.7	96.1	96.0	95.9
*τ* = 0, *N* = 50, *θ* = 1	94.9	95.7	95.7	95.6	96.0	95.9	96.0
*τ* = 0.5, *N* = 50, *θ* = 0	96.0	94.9	94.0	94.8	93.9	94.2	94.0
*τ* = 0.5, *N* = 50, *θ* = 0.5	95.5	94.6	93.8	94.6	94.2	94.1	94.1
*τ* = 0.5, *N* = 50, *θ* = 1	93.0	94.0	93.8	94.5	94.3	94.2	94.3
Standardized mean difference (Hedges’ *g*):							
*τ* = 0, *N* = 5, *θ* = 0	98.0	97.0	96.7	96.8	96.4	96.2	96.2
*τ* = 0, *N* = 5, *θ* = 0.5	97.7	96.8	96.6	96.6	96.4	96.2	96.4
*τ* = 0, *N* = 5, *θ* = 1	96.9	96.3	96.2	96.4	96.1	96.0	96.3
*τ* = 0, *N* = 50, *θ* = 0	97.6	96.7	96.5	96.1	96.2	95.8	95.8
*τ* = 0, *N* = 50, *θ* = 0.5	94.0	94.8	95.1	95.1	95.7	95.9	96.0
*τ* = 0, *N* = 50, *θ* = 1	81.2	89.0	92.1	93.4	94.9	95.6	95.9
*τ* = 0.5, *N* = 50, *θ* = 0	96.1	94.9	94.0	94.9	94.0	94.2	94.0
*τ* = 0.5, *N* = 50, *θ* = 0.5	91.6	93.1	93.3	94.4	94.2	94.1	94.1
*τ* = 0.5, *N* = 50, *θ* = 1	76.6	88.3	91.2	93.1	93.9	94.2	94.3
Log odds ratio:							
*τ* = 0, *N* = 5, *θ* = 0	98.2	97.3	97.0	96.9	96.5	96.4	95.9
*τ* = 0, *N* = 5, *θ* = 1	98.2	97.5	96.9	96.6	96.4	96.1	96.4
*τ* = 0, *N* = 5, *θ* = 1.5	97.8	97.8	97.2	96.9	96.4	96.5	96.2
*τ* = 0, *N* = 50, *θ* = 0	98.0	97.1	96.6	96.0	95.7	95.7	95.7
*τ* = 0, *N* = 50, *θ* = 1	94.9	96.1	95.9	96.0	95.7	95.8	95.7
*τ* = 0, *N* = 50, *θ* = 1.5	82.5	91.7	93.6	94.6	95.4	96.0	95.6
*τ* = 0.5, *N* = 50, *θ* = 0	97.7	96.5	95.7	95.2	94.6	94.2	94.3
*τ* = 0.5, *N* = 50, *θ* = 1	91.7	92.7	92.5	92.9	93.5	93.9	94.3
*τ* = 0.5, *N* = 50, *θ* = 1.5	75.0	83.5	84.7	86.7	90.4	93.7	94.1
Log risk ratio:							
*τ* = 0, *N* = 5, *θ* = 0	84.0	90.2	93.1	94.4	95.3	96.2	96.0
*τ* = 0, *N* = 5, *θ* = 0.3	86.2	89.9	92.0	93.0	94.8	95.8	96.1
*τ* = 0, *N* = 50, *θ* = 0	7.4	25.6	41.6	56.2	73.0	90.3	93.5
*τ* = 0, *N* = 50, *θ* = 0.3	9.7	25.1	38.2	49.9	66.5	87.6	92.4
Risk difference:							
*τ* = 0, *N* = 5, *θ* = 0	94.0	94.2	94.9	95.6	95.9	96.3	95.8
*τ* = 0, *N* = 5, *θ* = 0.2	94.4	94.5	94.7	95.0	95.6	96.0	96.0
*τ* = 0, *N* = 50, *θ* = 0	92.7	93.5	93.9	94.5	94.9	95.7	95.6
*τ* = 0, *N* = 50, *θ* = 0.2	92.4	93.3	93.2	93.9	94.5	95.6	95.7

Fig 1 and Table 1 indicate that the estimated MD was almost unbiased in all situations with different numbers of studies and different extents of heterogeneity, even if the studies had very small sample sizes. As the trends in the plots for Δ = 0.5, 1, 2, and 5 were fairly similar to those for Δ = 0, they were not displayed in Fig 1 due to space limit. Table 2 shows that the CI coverage probability of the MD was fairly close to the nominal confidence level 95% in most cases. The coverage was slightly below 95% when the number of studies was small (*N* = 5) and the sample sizes were also very small (between 5 and 10) within studies.

When the true SMD was zero in the left panels of Fig 2, both Cohen’s *d* and Hedges’ *g* were almost unbiased. The box of Cohen’s *d* was slightly larger vertically than that of Hedges’ *g* when the sample sizes within studies were small, so the point estimates of Hedges’ *g* were more concentrated around the true SMD. The CI coverage was also close to the nominal 95% level. However, as the true SMD increased from 0 to 0.5 and to 1, both Cohen’s *d* and Hedges’*g* began to have bias, and the bias increased as the sample sizes decreased within studies. Cohen’s *d* generally produced less bias in the estimated overall SMD than Hedges’ *g*, as shown in Table 1. The CI coverage of Cohen’s *d* was still close to 95% when the true SMD increased, but that of Hedges’ *g* dropped below 80% when the sample size was fairly small (between 5 and 10), the true SMD was fairly large (*θ* = 1), and the number of studies was large (*N* = 50).

The patterns in Fig 3 of the ORs for binary outcomes were similar to those in Fig 2. The estimated overall log ORs were almost unbiased when the true log OR was zero. As the true log OR increased to 1 and to 1.5 and the sample sizes within studies decreased, the bias in the estimated overall log OR tended to be larger in the negative direction. Also, the CI coverage dropped dramatically when the number of studies and the between-study variance were large in Table 2. For example, when *τ* = 0, *N* = 5, *θ* = 1.5, and the sample size of each study was between 5 and 10, the bias of the estimated overall log OR was −0.30 and the CI coverage was 97.8%. The log OR underestimated the true value *θ*. Among the simulated meta-analyses whose CIs did not cover *θ*, 2.2% had CIs entirely below *θ*, while only one meta-analysis (0.01%) had a CI entirely above *θ*. As the number of studies increased to *N* = 50 and other parameters unchanged, the bias was still −0.30, but the CI coverage decreased to 82.5%. The CIs of the meta-analyses not covering *θ* were all below *θ*. Therefore, the low CI coverage was likely because the CI became shorter as the number of studies *N* increased while the bias remained.

Compared with the log OR, the log RR in Fig 4 was more sensitive to the sample sizes within studies. The estimated overall log RR had tiny bias and its CI coverage was close to 95% when the sample sizes within studies were large (more than 500). However, the bias was substantial and the CI coverage was fairly low even when the sample sizes were moderate (between 50 and 100). Like the situation for the log OR, the poor CI coverage for the log RR related to the bias. For example, when *τ* = 0, *N* = 5, *θ* = 0.3, and the sample size of each study was between 5 and 10, the bias of the estimated overall log RR was 0.26 and the CI coverage was 86.2%. The log RR overestimated the true value *θ*. The CIs of the simulated meta-analyses not covering *θ* were all above *θ*. When *N* increased to 50 and other parameters unchanged, the bias was 0.35 and the CI coverage dropped dramatically to 9.7%. The CIs of the simulated meta-analyses not covering *θ* were also all above *θ*.

Fig 5 shows that the estimated overall RD was almost unbiased when the true RD was zero and had small bias when the true RD was 0.2. The bias was relatively large when the sample sizes within studies were fairly small. The CI coverages were between 92% and 96% in all situations.

In addition, Figures A–F in S1 File present scatter plots of the sample effect sizes against their precisions (i.e., the inverse of their sample variances) in ten selected simulated meta-analyses with small sample sizes for the MD, SMD (including both Cohen’s *d* and Hedges’ *g*), log OR, log RR, and RD. They are plotted using the same idea of the funnel plot for assessing publication bias [56], and they roughly illustrate the association between the sample effect sizes *y*_*i*_ and their within-study variances $s_{i}^{2}$. Figure A in S1 File indicates that this association seemed tiny for the MD, which was consistent with our conclusion that the MD *y*_*i*_ and its variance $s_{i}^{2}$ are independent in theory. The other figures show different extents of association for the SMD, log OR, log RR, and RD. For example, the estimated SMDs that were closer to zero tended to have larger precisions (i.e., smaller variances) in Figures B and C in S1 File.

## Discussion

This article has shown that the bias in the overall estimates of the SMD, log OR, log RR, and RD may be substantial in meta-analyses with small sample sizes. The estimated overall MD was almost unbiased in nearly all simulation settings, mainly because its point estimate and within-study variance were independent. However, for the other four effect sizes except the MD, the intrinsic association between their point estimates and estimated variances within studies may be strong, so the meta-analysis results were biased in many simulation settings. Therefore, when the collected studies have small sample sizes, researchers need to choose a proper effect size and perform the meta-analysis with great cautions.

Surprisingly, to estimate the overall SMD, using Cohen’s *d* led to noticeably less bias than using Hedges’ *g* in our simulation studies, although Hedges’ *g* was designed as a bias-corrected estimate of the SMD within individual studies. For example, in one of our simulated fixed-effect meta-analyses with 50 studies and 5 to 10 samples in each study (the true SMD was 1), the average of Cohen’s *d* in the 50 studies was around 1.29, while the average of Hedges’ *g* 1.07 was closer to the true value 1. This was consistent with the fact that Hedges’ *g* was generally less biased within individual studies. However, the meta-analytic overall Cohen’s *d* was 0.98, which was much closer to 1 compared with the meta-analytic overall Hedges’ *g* 0.89, because of the sampling error in these effect sizes’ variances that caused the association between the effect sizes and the variances. Note that, instead of advocating that Cohen’s *d* is always preferred than Hedges’ *g* in meta-analyses, this article only reminds researchers that Cohen’s *d* may be less biased in at least some meta-analytic results, and the argument for the use of Hedges’ *g* in the presence of small sample sizes needs to be carefully examined.

In addition, there are alternative methods to estimate the within-study variance of Hedges’ *g* besides the one used in our article. Specifically, our simulation studies used $s_{gi}^{2}=\frac{1}{n_{i0}}+\frac{1}{n_{i1}}+\frac{g_{i}^{2}}{2(n_{i0}+n_{i1})}$, where *g*_*i*_ is the point estimate of Hedges’ *g* in study *i*; this calculation was introduced on page 86 in Hedges and Olkin [14]. Recall that Hedges’ *g* is calculated by multiplying Cohen’s *d* by a bias-correction coefficient; that is, *g*_*i*_ = *J*_*i*_*d*_*i*_, where $J_{i}=1-\frac{3}{4(n_{i0}+n_{i1})-9}$ and *d*_*i*_ is the point estimate of Cohen’s in study *i*. Therefore, the variance of Hedges’ *g* can be alternatively estimated as $s_{gi}^{2}=J_{i}^{2}s_{di}^{2}$, where $s_{di}^{2}$ is the within-study variance of Cohen’s *d*; see, e.g., page 226 in Cooper et al. [34]. Using this alternative calculation for the within-study variances of Hedges’ *g*, the combined SMD may remain biased. For example, consider a special case that all *N* studies in a meta-analysis have the same sample size *n*, so the bias-correction coefficients in all studies are equal: *J*_*i*_ = *J*. Using the fixed-effect model, the expectation of the combined Cohen’s *d* is

$$\mu_{d}=E\;[\frac{\sum d_{i}/s_{di}^{2}}{\sum 1/s_{di}^{2}}],$$

and the expectation of the combined Hedges’ *g* is

$$\mu_{g}=E\;\left[\frac{\sum g_{i}/s_{gi}^{2}}{\sum 1/s_{gi}^{2}}\right]=E\;\left[\frac{\sum (Jd_{i})/\left(J^{2}s_{di}^{2}\right)}{\sum 1/\left(J^{2}s_{di}^{2}\right)}\right]=J\mu_{d}.$$

Because *J* is a coefficient always less than 1, we have *μ*_*g*_ < *μ*_*d*_ if assuming *μ*_*d*_ is positive. If the true overall SMD *θ* is also positive and the combined Cohen’s *d* underestimates it (as in our simulation studies), then *μ*_*g*_ < *μ*_*d*_ < *θ*, indicating that the combined Hedges’ *g* is more biased. However, if the combined Cohen’s *d* overestimates the overall SMD (i.e., *μ*_*d*_ > *θ*), then the combined Hedges’ *g* might be less biased.

This article helps explain the phenomenon of the inflated type I error rates for testing for publication bias. To detect potential publication bias in meta-analyses, it has been popular to check for the association between the study-specific effect sizes and their standard errors using the funnel plot or Egger’s regression test [15]. However, it is well known that such association may be intrinsic for binary outcomes even if no publication bias appears, so Egger’s test may have an inflated type I error rate [31, 32]. In addition to the intrinsic association for binary outcomes, this article indicates that such a problem also exists when using the SMD for continuous outcomes. Although the meta-analyses with false positive results do not truly have publication bias, they may still suffer from bias due to sampling error.

Moreover, our findings imply that the magnitude of sample size may not be viewed as an absolute concept in meta-analyses; we may not determine whether a sample size is small or large without taking other parameters into account. For example, using the log OR as the effect size, Fig 3(A), 3(D) and 3(G) show that a sample size of 10 to 20 may be large enough to produce desirable meta-analysis results when the true log OR is zero. However, when the heterogeneity, the number of studies, and the true log OR are large, Fig 3(I) shows that a sample size of 50 to 100 may not be adequate.

The bias of the estimated overall log RR was particularly substantial in Fig 4; this may be related to the effect of the weighting bias for binary outcomes [57]. However, unlike the purpose of Tang [57], this article focused on the bias completely due to sample error which exists for both continuous and binary outcomes.

This article performed the simulated meta-analyses using the popular inverse-of-variance method in a frequentist way. Alternatively, several exact models have been proposed for binary outcomes; they do not require the normal approximation to estimate the study-specific effect sizes and their within-study variances [58–62]. The event numbers in the compared groups can be directly modeled as binomial distributions, thus accounting for sampling error in both point estimates of effect sizes and their variances. Similar exact models are also needed for continuous outcomes to avoid treating the within-study variances as if they were the true variances; we leave them as future work.

## Supporting information

S1 File Scatter plots of the sample effect sizes against their precisions (i.e., the inverse of their sample variances) in some simulated meta-analyses with small sample sizes. (PDF)

S2 File R code for the simulation studies. (ZIP)

S3 File Simulation results for the mean difference. (ZIP)

S4 File Simulation results for the standardized mean difference. (ZIP)

S5 File Simulation results for the log odds ratio. (ZIP)

S6 File Simulation results for the log risk ratio. (ZIP)

S7 File Simulation results for the risk difference. (ZIP)
