# Supplementary material for: Bias caused by sampling error in meta-analysis with small sample sizes
Source: PLoS One. 2018 Sep 13;13(9):e0204056. doi: 10.1371/journal.pone.0204056 (PMC6136825; doi:10.1371/journal.pone.0204056)
Supplement: S1 File — (PDF) [file pone.0204056.s001.pdf]

Supporting information for

“Bias caused by sampling error in meta-analysis with small sample sizes”

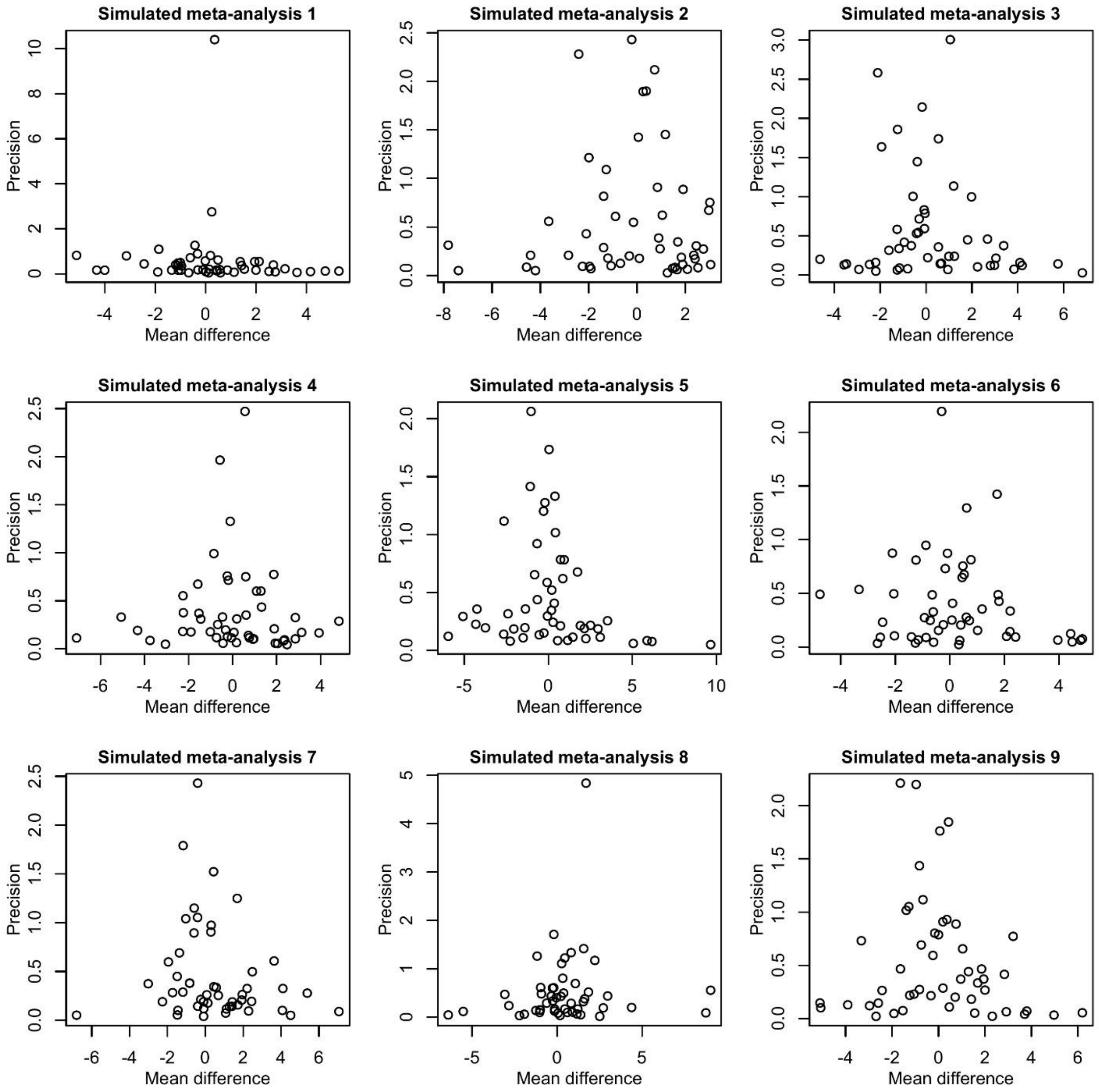

Figure A. Scatter plots of the sample mean differences against their precisions (the inverse of their sample variances) in the first ten simulated meta-analyses with $\tau = 0$, $N = 50$, $\Delta = 0$, and sample sizes between 5 and 10.

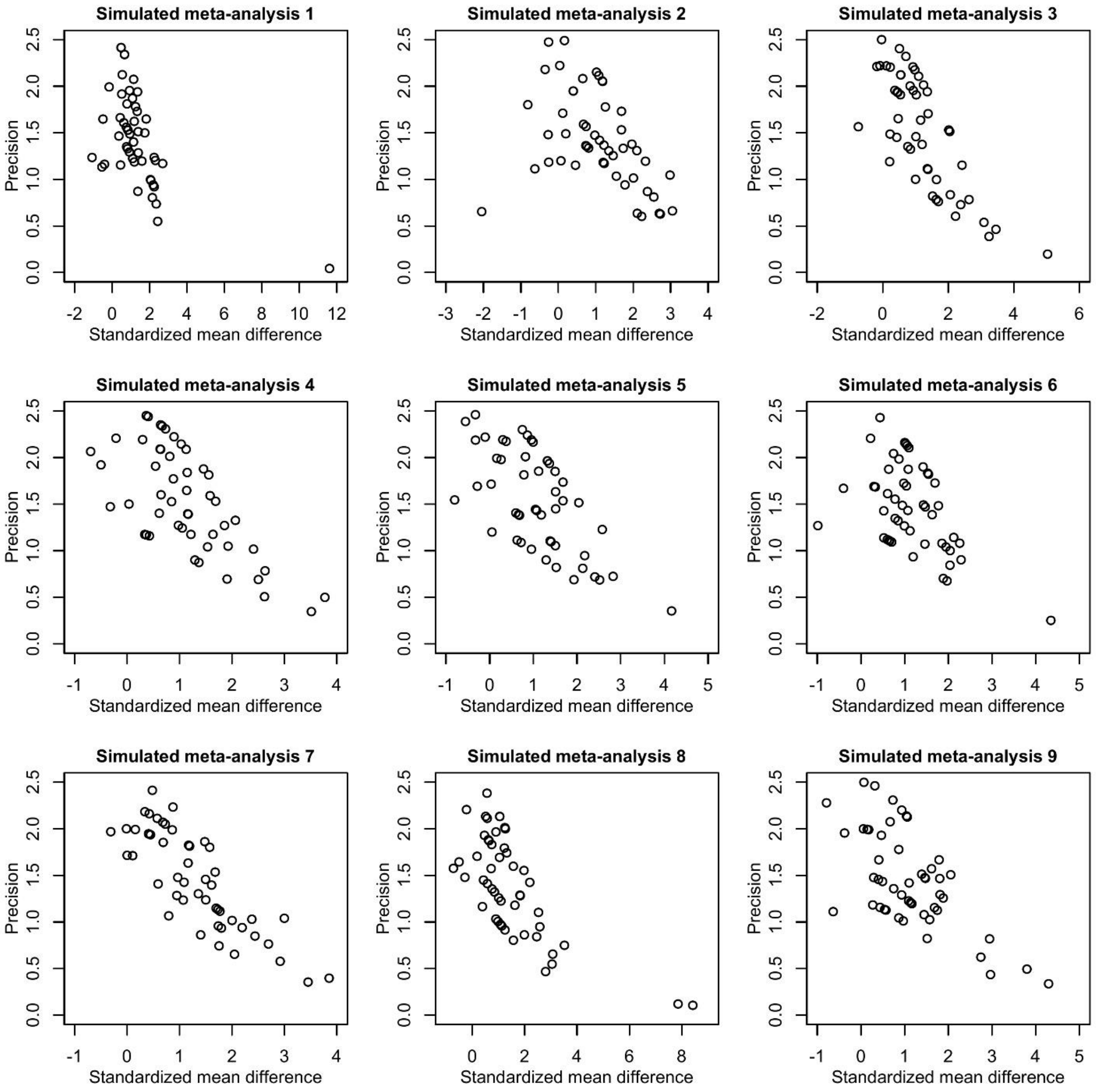

Figure B. Scatter plots of the sample standardized mean differences estimated using Cohen's d against their precisions (the inverse of their sample variances) in the first ten simulated meta-analyses with $\tau = 0$, $N = 50$, $\theta = 1$, and sample sizes between 5 and 10.

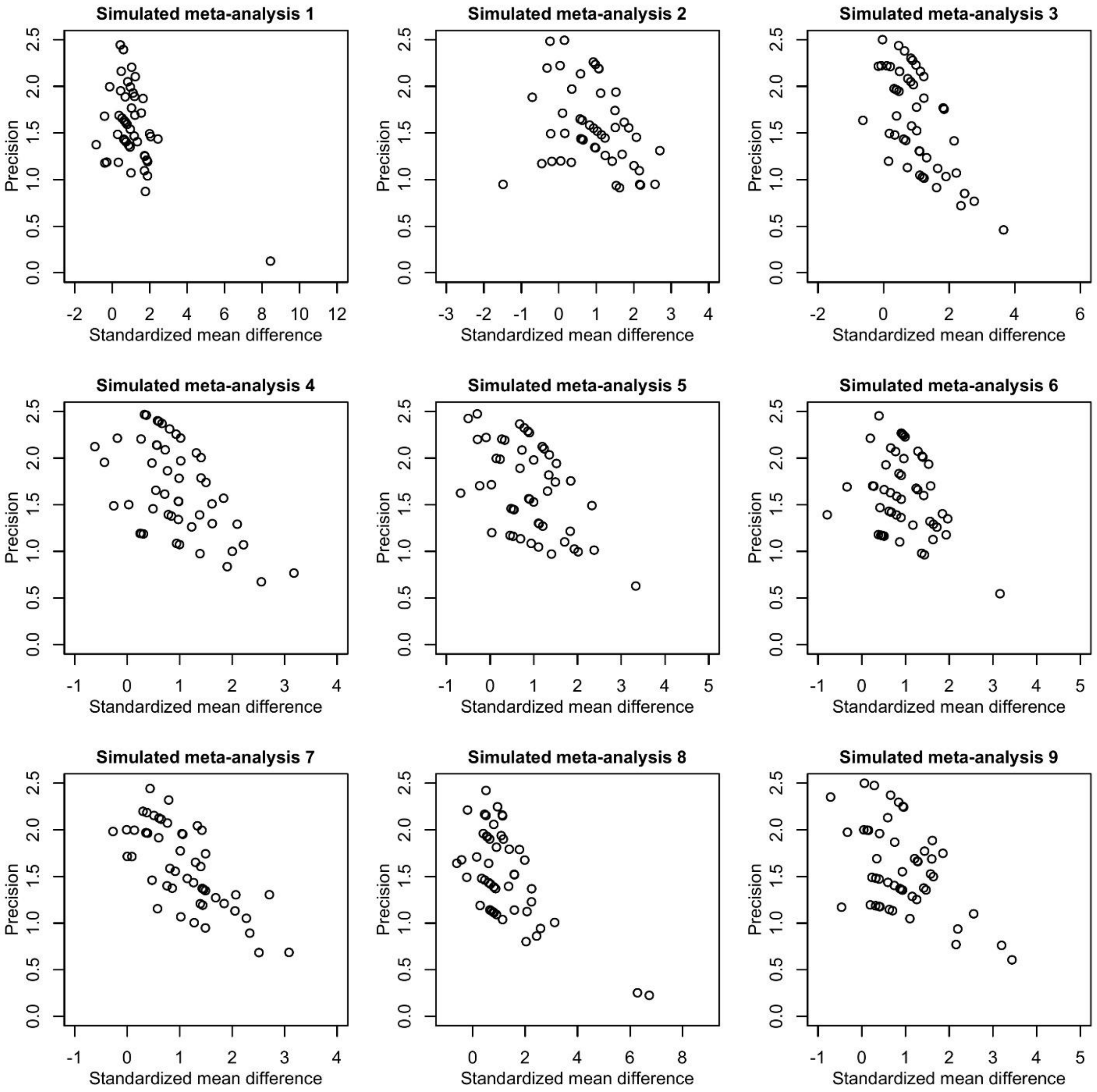

Figure C. Scatter plots of the sample standardized mean differences estimated using Hedges' g against their precisions (the inverse of their sample variances) in the first ten simulated meta-analyses with τ = 0, N = 50, θ = 1, and sample sizes between 5 and 10.

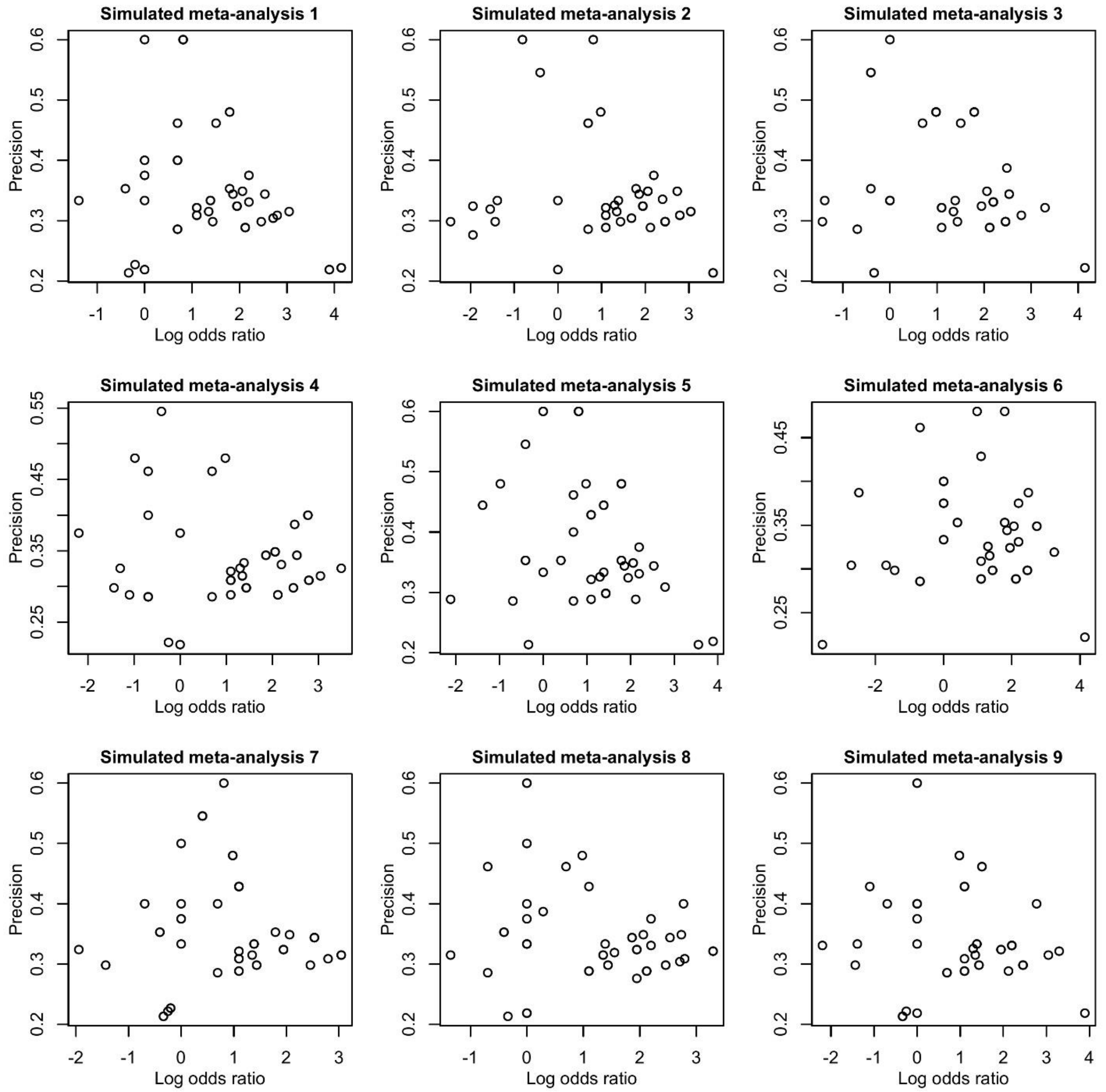

Figure D. Scatter plots of the sample log odds ratios against their precisions (the inverse of their sample variances) in the first ten simulated meta-analyses with $\tau = 0$, $N = 50$, $\theta = 1.5$, and sample sizes between 5 and 10.

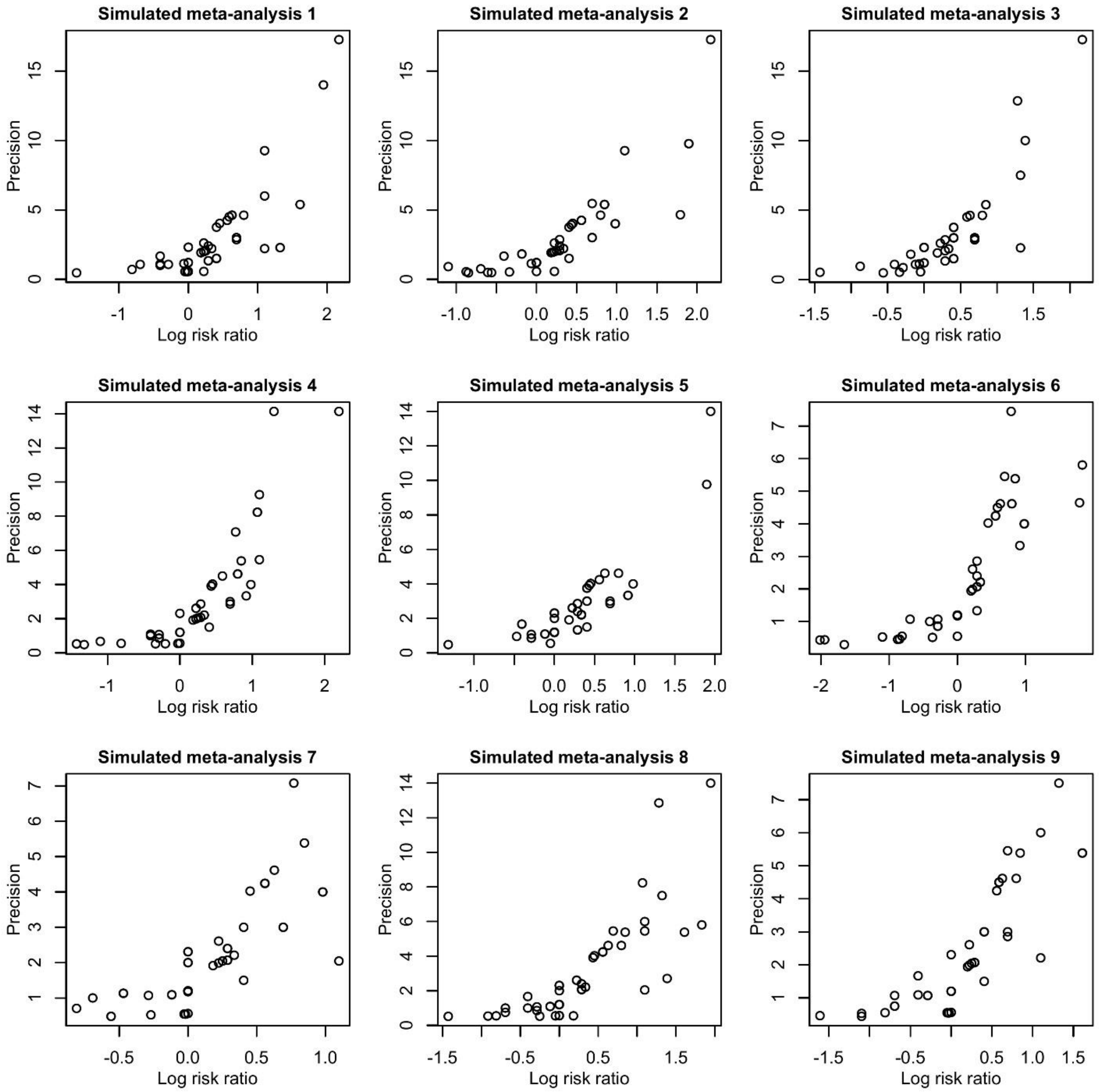

Figure E. Scatter plots of the sample log risk ratios against their precisions (the inverse of their sample variances) in the first ten simulated meta-analyses with $\tau = 0$, $N = 50$, $\theta = 0.3$, and sample sizes between 5 and 10.

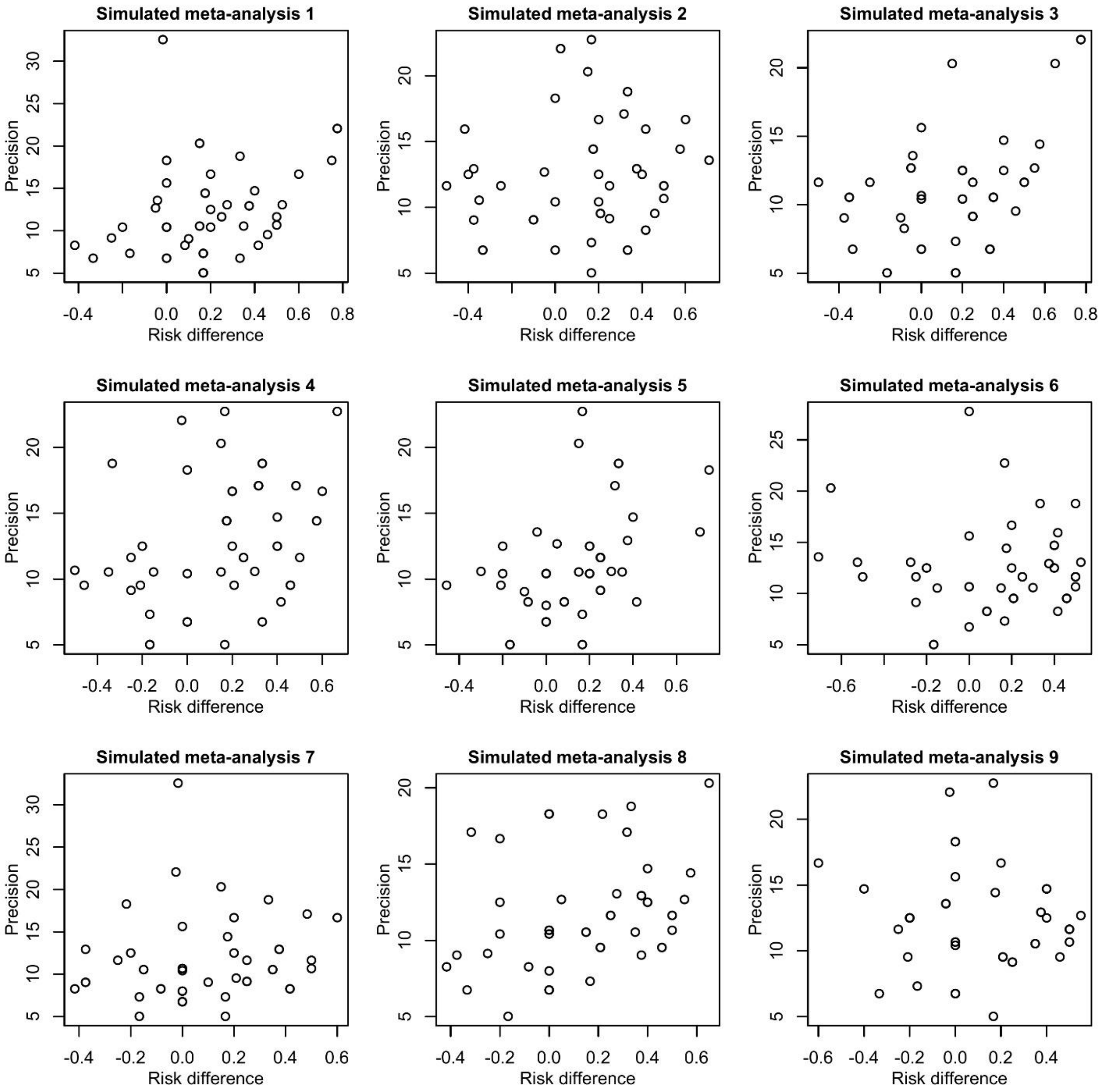

Figure F. Scatter plots of the sample risk differences against their precisions (the inverse of their sample variances) in the first ten simulated meta-analyses with $\tau = 0$, $N = 50$, $\theta = 0.2$, and sample sizes between 5 and 10.
